# Educational potential of a virtual patient system for caring for traumatized patients in primary care

**DOI:** 10.1186/1472-6920-13-110

**Published:** 2013-08-19

**Authors:** Solvig Ekblad, Richard F Mollica, Uno Fors, Ioannis Pantziaras, James Lavelle

**Affiliations:** 1 Department of Learning Informatics, Management and Ethics (LIME), Cultural Medicine Unit, Karolinska Institutet, Tomtebodavägen 18A, plan 3, SE-171 77, Stockholm, Sweden; 2 Department of Computer and Systems Sciences, Stockholm University, Stockholm, Sweden; 3 Harvard Program in Refugee Trauma (HPRT), Massachusetts General Hospital, Boston, MA, USA

**Keywords:** Primary health care, Virtual patients, Virtual encounters, Refugees, PTSD, Depression

## Abstract

**Background:**

Virtual Patients (VPs) have been used in undergraduate healthcare education for many years. This project is focused on using VPs for training professionals to care for highly vulnerable patient populations. The aim of the study was to evaluate if Refugee Trauma VPs was perceived as an effective and engaging learning tool by primary care professionals (PCPs) in a Primary Health Care Centre (PHC).

**Methods:**

A VP system was designed to create realistic and engaging VP cases for Refugee Trauma for training refugee patient interview, use of established trauma and mental health instruments as well as to give feedback to the learners. The patient interview section was based on video clips with a Bosnian actor with a trauma story and mental health problems. The video clips were recorded in Bosnian language to further increase the realism, but also subtitled in English. The system was evaluated by 11 volunteering primary health clinicians at the Lynn Community Health Centre, Lynn, Massachusetts, USA. The participants were invited to provide insights/feedback about the system’s usefulness and educational value. A mixed methodological approach was used, generating both quantitative and qualitative data.

**Results:**

Self-reported dimensions of clinical care, pre and post questionnaire questions on the PCPs clinical worldview, motivation to use the VP, and IT Proficiency. Construct items used in these questionnaires had previously demonstrated high face and construct validity. The participants ranked the mental status examination more positively after the simulation exercise compared to before the simulation. Follow up interviews supported the results.

**Conclusions:**

Even though virtual clinical encounters are quite a new paradigm in PHC, the participants in the present study considered our VP case to be a relevant and promising educational tool. Next phase of our project will be a RCT study including comparison with specially prepared paper-cases and determinative input on improving clinical diagnosis and treatment of the traumatized refugee patient.

## Background

At the end of 2011, forcibly displaced people worldwide exceeded 42 million. This figure has been about the same during the last fifth consecutive years (2007–2011). This figure included 26.4 million internally displaced persons (IDPs), 15.2 million refugees, 10.4 million under UNHCR’s mandate, 4.8 million Palestinian refugees and 895,000 asylum seekers [1]. In the refugee group, about half are women, nearly half are children under the age of 18 and they live mainly in low-income countries, close to wars and mass violence, and they are not yet resettled. In 2010, it was estimated that there were 40 million foreign-born people or 12.9 percent of the total population living in the United States [2]. Since 1975, the U.S. has resettled over 3 million refugees from all over the world [3]. Most immigrants and all refugees come to the US to escape poverty, mass violence and/or political oppression. Immigrants and refugees in the US have been demonstrated to have major issues in seeking primary health care leading to significant problems in health disparities [4-17].

However, primary care practitioners (PCPs) have limited training in identification and treatment of mental health problems such as depression and Posttraumatic Stress Disorders (PTSD) [18], which calls for improved training on the management of traumatized patients in both undergraduate and postgraduate education. The Harvard Program in Refugee Trauma (HPRT) at Massachusetts General Hospital and Harvard Medical School has been a pioneer over the past 30 years in the identification and treatment of highly traumatized refugee patients from culturally diverse backgrounds [19].

### Traumatic life events and health

Evidence shows that trauma is a risk factor for both physical and mental health. Exposure to traumatic life events has been demonstrated to be highly correlated with smoking mortality, an increase in alcohol abuse, drug use and direct physical health problems as well as other long term physical illnesses [20]. It has been well established that cumulative trauma is associated with the psychiatric diagnosis of PTSD and depression in a dose-effect relationship, i.e. increasing levels of trauma lead to higher rates and severity of PTSD and depression [21,22]. Over the past 25 years major community studies [23] have demonstrated the high rates of PTSD, depression and physical disability in highly traumatized refugee populations [23-26]. Mental health and physical illness are directly related to major lifestyle factors such as diet, smoking, obesity, lack of exercise and alcohol/substance abuse that can be directly managed and improved in the primary health care (PHC) setting and through community-based interventions. Therefore, PCPs need to receive training how to accurately identify trauma as a major medical and mental health risk factor.

### Virtual patients as a training model

Virtual Patients (VPs) are interactive computer simulations of patient encounters used in health care education. The VP is a virtual representation of a patient encounter for learning and assessment, typically including interactive features for illness history taking, physical examination, laboratory tests as well as features for suggesting diagnosis and treatment plan [27,28]. According to situated learning theory, students can acquire knowledge by being engaged in tasks that in an authentic way parallel real world activities [29]. Communication research has shown that highly engaging and challenging interactive media interfaces can promote deeper motivation and concentration [30]. Those factors have a major significant influence on the way we interact with others, eg. authentic patient encounters and how we interpret verbal and non-verbal communication cues. Research in education has reported that emotional mediated experiences have a positive impact on cognitive learning outcomes [31,32]. Thus, they lead to heightened involvement and decreased cognitive overload. Authenticity is critical to whether a virtual patient can be considered to be part of a situated learning endeavor, indicating that VPs may provide reliable, valid, and applicable representations of live patients [33]. VPs can emulate a problem-based learning environment to assist medical practitioners in active, independent (and group) learning and problem-solving and are also giving the learner automatic feedback on the patient management process.

VPs have been shown to have a great educational value especially for training clinical reasoning [34]. Virtual patients are also shown to have a potential to emphasize socio-cultural aspects and cultural differences as they pertain to healthcare education [28,35]. The use of VPs has been proven to be effective in the training of surgeons [36], medical students [27,37,38] as well as psychology students [39,40]. Research into the use of VPs in psychotherapy training is quite new [41]. Little is known about the efficacy of the VP in training PCPs for traumatized refugee populations and for patients with psychiatric symptoms, although an evaluation by medical students of a VP with PTSD in an adolescent female has been published [42].

VPs may offer a holistic approach to medical education using a model that emphasizes both the patient and the doctor’s perspective. Therefore VPs might have a potential to dramatically increase skills and knowledge as well as foster trust, respect and empathy in the doctor-patient relationship.

Our team has performed a pilot study in Sweden with VPs for training the management of treating culturally diverse and traumatized refugees [43]. That study indicated that the Refugee trauma VP was well received by the participants and that they appraised it as depicting in a realistic way a real life doctor–patient encounter and having a good potential for training patient management of mentally traumatized refugees.

In this study, we have set up an international American-Swedish collaboration, aiming to further develop the initial Refugee trauma VP case system and investigate the potential of that system to train PCPs in the management of culturally diverse, highly traumatized refugee patients with co-morbid health and mental health problems.

## Methods

### Refugee trauma system

The initial prototype is designed in such a way that it makes it possible for user interaction in the following areas of medical care: (1) medical interview, including a comprehensive illness history dialogue with the virtual patient for investigating the chief complaint, history of the present illness, and social history; (2) physical examination (including mental status examination); (3) screening instruments, including the Harvard Trauma Questionnaire (HTQ) and the Hopkins Symptom Checklist (HSCL-25); (4) laboratory tests and imaging studies; (5) additional data (i.e. information about the country of origin, laws about migration in the host country and links to relevant sources of information in the world wide web); and (6) preliminary assessment (i.e., treatment plan). The preliminary system included open-ended questions which make it possible for the participant to present a structured summary of the patient’s history, a probable diagnosis and a summary of a plan for treatment. An automated and individualized feedback upon actions taken, their relevance and the quality of case management appears after this module. The content of this feedback include a list of activities performed during the medical examination (e.g. questions asked, ordered laboratory tests and examination of the virtual patient) followed by a comment on its relevance. Further, a summary of the case management as suggested by a domain expert is presented to the user, followed by feedback from a “virtual advisor” (VA).

The Refugee trauma pilot study [43] was redesigned to increase the realism and engagement in the patient interview, incorporate established trauma and mental health instruments as well as to facilitate and improve the feedback to the learners. Proposals by the participants of the Swedish pilot study were taken thoroughly in consideration in order to achieve this.

**Figure 1:**
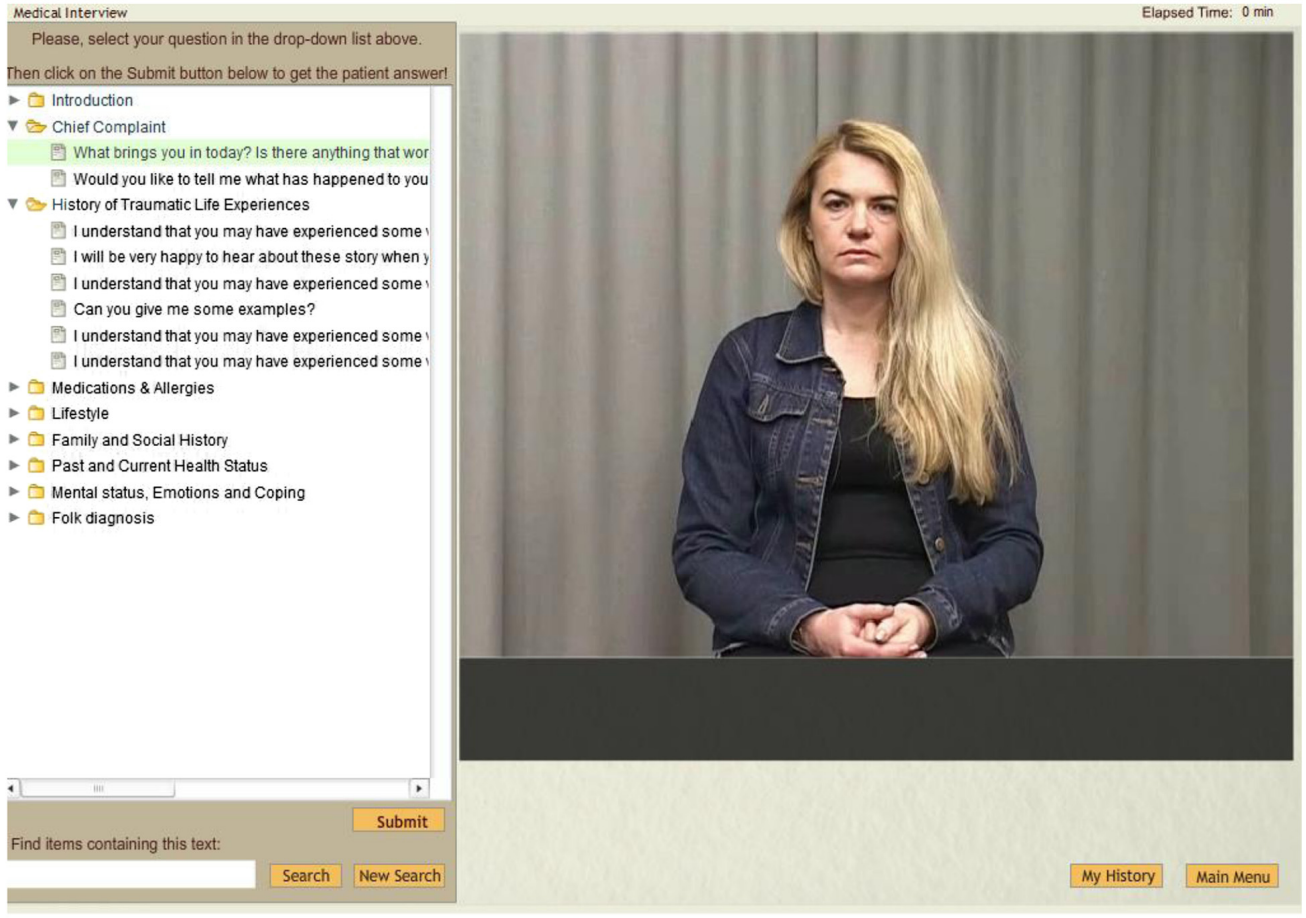
**History-taking section.** The interview section where the user can ask the VP questions regarding her trauma story and receive answers in terms of video clips.

The patient interview section was in this new version based on video clips with a Bosnian actor who agreed to be interviewed as a realistic appearing Bosnian refugee with a trauma story and mental health problems (see Figure 1). The video clips were recorded in Bosnian language to further increase the realism, but also subtitled in English. This new system was called the Refugee Trauma Simulator, or RTSim.

**Figure 2:**
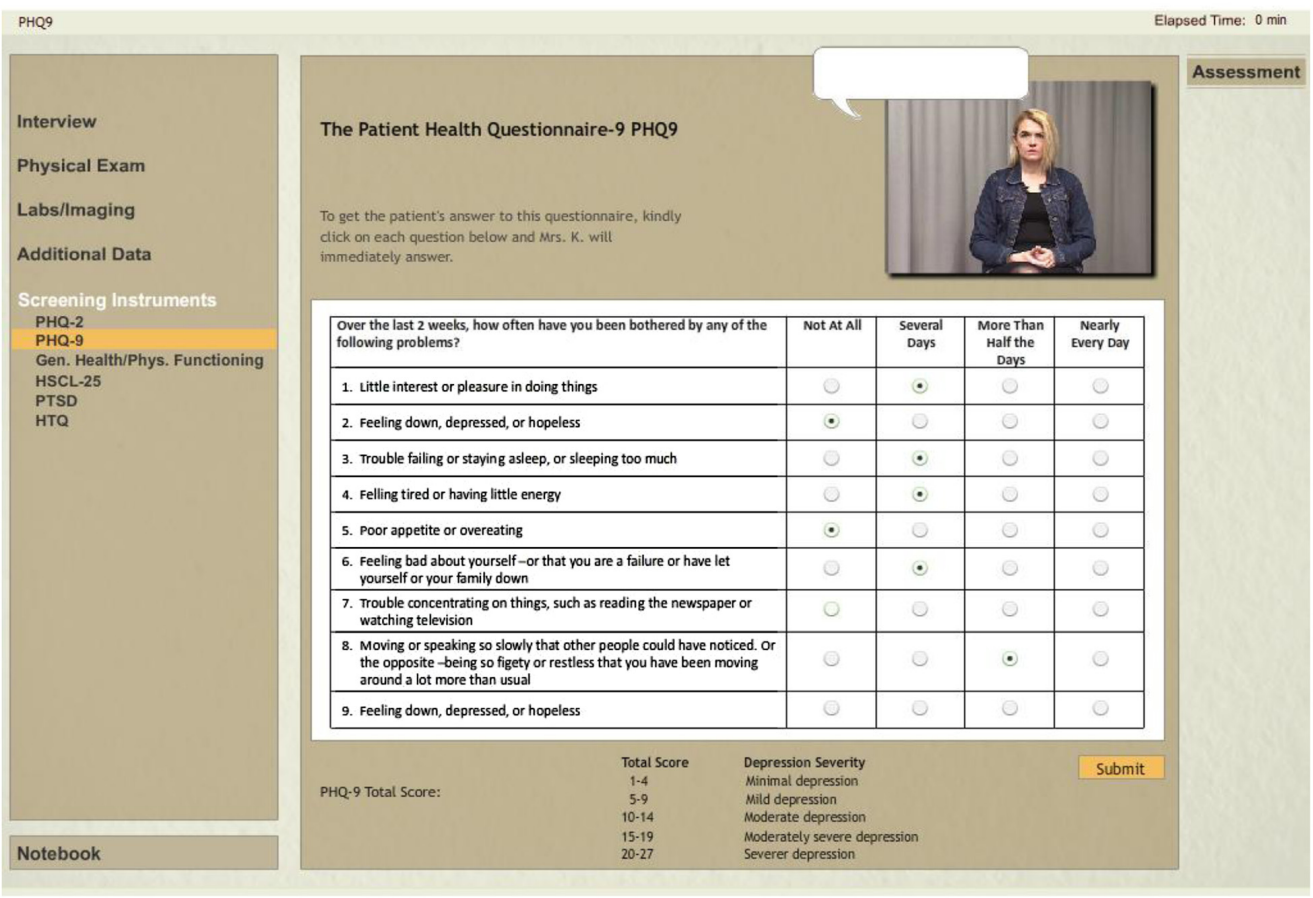
**Screening instruments.** The user can fill in any of the six available instruments.

The system also enables the user to train to select and fill in appropriate trauma and mental health instruments like PHQ-2, PHQ-9, PTSD, HTQ, HSCL-25 and others (see Figure 2).

**Figure 3:**
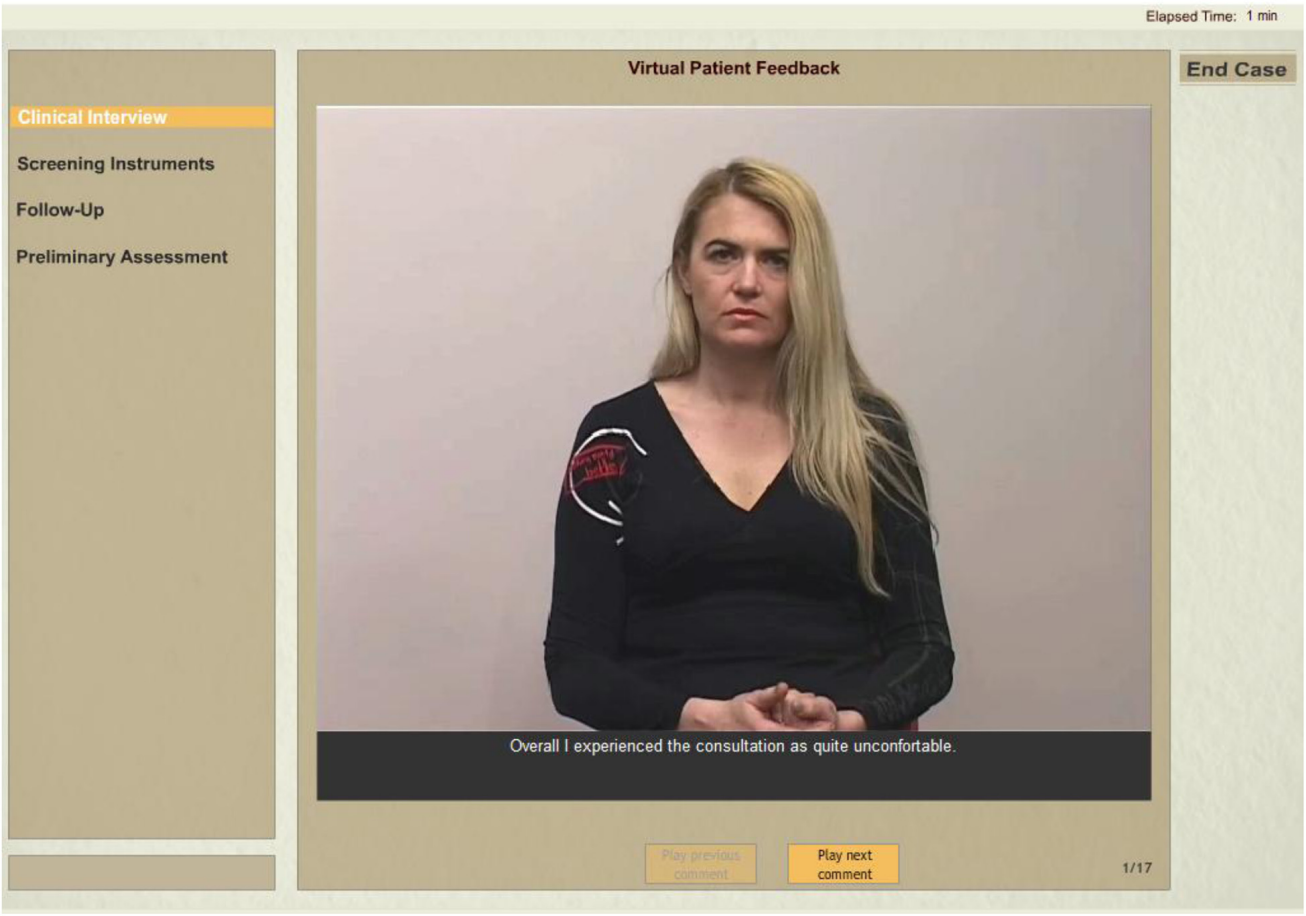
**Feedback section.** After the patient examination and the suggested patient management issues, the user receives feedback from both the refugee herself (above) and a virtual advisor/expert.

After the interaction with the virtual patient is over (including actions as suggesting possible revisits, referrals, setting diagnoses and suggesting a treatment plan) the user receives feedback by the virtual refugee herself. Following this, a virtual advisor (domain expert) also gives his/her automated and individualized advice to the user to further increase the learning outcomes (see Figure 3).

### Study design

11 PCPs at the Lynn Community Health Centre (LCHC), Lynn, Massachusetts, were invited to participate in the study and all of them volunteered to be included in the study. The study was performed in three steps. The first step started with giving informed consent to the participants. After the invited participants had returned the signed consent form, they got access to an on-line version of the actual VP-system. In session 1, the PCPs received an introduction to the VP system and they were administered a pre-test questionnaire regarding their IT Proficiency, overview of clinical worldview and current motivation to use VPs for training.

In Session 2, all PCPs run the system on their own and were asked to encounter and manage the virtual refugee as if it had been a real patient. Upon completion of this session all PCPs received a post-test questionnaire.

One month later, after the PCPs had some time to reflect on their experience, they were invited for Session 3, a telephone interview. During the interview they were able to reflect on the quality and usefulness of the Refugee trauma VP and made recommendations for further improvements of the system.

Written informed consent for participation in the study was obtained from the participants. This study was approved by the IRB at Massachusetts General Hospital (IRB protocol number 2011P001736). Attention was paid to all ethical considerations during the recruitment procedure of the participants and at each step during preparation, performance and data analysis, including consent form and information sheet.

### The setting

The Institute of Medicine defines primary health care as “integrated and accessible care by clinicians who are responsible for addressing a majority of personal health needs through a sustained partnership with patients and practicing in a family and community context” [44]. PHC is therefore considered an ideal health care environment of addressing the health and mental health needs of traumatized persons from culturally diverse communities. Primary health care, for example, serves as the initial point of contact for patients with health related trauma problems, depression and PTSD [45]. Yet, the usual care by PCPs may be less than optimal with studies indicating the recognition of trauma-related distress as less than 40% [46], diagnosis of PTSD as low as 2% [46] and depression less than 50% [47]. In primary health care veteran clinics where PTSD and depression should be routinely diagnosed, less than 50% of diagnosable patients were identified [48]. In a Swedish study at a psychiatric outpatient clinic in a suburb south of Stockholm in a multicultural immigrant/refugee population only 40% of the diagnosable patients were recognized with diagnosis of PTSD [49]. Under-diagnosis and under-treatment for historically disadvantaged ethnic groups may be especially high [50-52]. Despite the inherent and current limitations of primary care, such as its fast-pace and time constraints, it remains the ideal place for diagnosis and treatment of health and mental health problems and primarily health care can be less stigmatizing than special mental health clinics and despite the many barriers, can meet the immigrants’ entire spectrum of mental health and medical needs.

### Participants

The PCPs were randomly selected from the entire list of 55 PCPs at LCHCs, however professionals that reported that they were not motivated in learning to use the VPs or stated that they had “poor” internet skills were excluded from being invited. That sample size allowed for intensive discussion and feedback with the PCP study group. All PCPs had previously been encountering traumatized refugees, but none was considered as a domain expert.

The 11 selected PCPs participated in the three 60 to 75 minute sessions led by the HPRT team leaders and received a US$ 225.00 dollar honorarium for each session. The participants were middle career practitioners (5 men and 6 women).

All out of the 11 initial PCPs completed the study and returned completed questionnaires. One of the initial PCPs was unable to take part to the follow up interview due to illness.

### Data collection

(1) Questionnaires

The pre and post questionnaires included survey questions on the PCP’s IT proficiency, clinical worldview, and current motivation to use VPs in the learning exercise. A similar questionnaire set was also used in the Swedish pilot study [43] with good results, making us believe that the questions had acceptable validity. The pre-test version included two parts. The first (“Overview of Clinical Worldview”) with the aim to examine the participants’ self-reported important issues during a “real life” medical examination, on a scale from 1 (no emphasis) to 5 (full emphasis). It consists of 10 items, divided into two parts; regarding the level of emphasis the clinician usually places on (a) data (chief complaint, history of present illness, physical exam, mental status exam, laboratory tests, and traditional healing exam) collected during the medical examination and (b) root causes of the disease (biological, psychological, social/economic and spiritual).

The second part of the pre-test questionnaire had the aim to examine the current motivation to use the VP before the simulation exercise on a 4-point Likert scale (1 = highly disagree, 4 = highly agree) with 17 questions (eg.” I am motivated to use VP as it helps to improve interdisciplinary communication”, “I believe that VP will help me to provide better care to my traumatized patients from any cultural background”).

(2) A follow-up interview

A follow-up interview by telephone or face-to-face was performed by fifth author (JL) after one month to receive more insights into each participant’s learning experience and attitudes toward the VP system. These were opened ended questions regarding the participant’s perception of educational potential and usefulness of the VP case, engagement perception, empowerment, virtual interpreter, virtual advisor and feedback.

### Data analysis

We quantified all findings from the answers in the questionnaires in the pilot study using SPSS 19.0. Analysis of the quantitative data were primarily descriptive since the small N = 11, does not allow for significance testing and will not allow for generalizability of gender and age. The analysis from the pre and post data included item-by item measures and median values for measuring the average rating of the Likert scale questions included in the revised KI-VP-LEQ. Focus was on an evaluation of the realistic nature and usefulness of the VP-system when analyzing the participants’ answers. Evaluation of the face validity, defined as “the extent to which the examination resembles real life situations” [53,54] was analysed in the issue of acceptance of the degree of the realistic nature of the patient simulation in relation to the actual task as it has been used before including the Swedish pilot study [43].

The qualitative data included the answers from the participants during the 10 telephone interviews lasting between 15 and 30 minutes. The data was analyzed according to inductive content analysis based on Graneheim and Lundman’s model [55]. Anonymous citations will be used to exemplify the quantitative data. Sometimes the grammar has been changed to increase readability, but in a way that the content has not been changed.

## Results

All 11 participants reported long experience of using computers in their daily clinical work and were able to run the RTSim system without any major problems. None of them mentioned that they had used virtual patients before.

### Overview of clinical worldview

Table 1 shows the self-reported dimensions of clinical care, pre-test and post-test on the questionnaires. The participants clearly ranked the mental status examination more important after the simulation exercise compared to before the simulation. There were minor differences regarding increased importance of Social and Spiritual root causes after the simulation exercise.

**Table 1:** Self-reported dimensions of clinical care (pre-test questionnaire and post-test questionnaire) ranked by level of emphasis (1 = no emphasis; 5 = full emphasis)

**Variables**	**Pre-test**	**Post-test**
	**All (N = 11)**	**All (N = 11)**
**Collected data**	**Median (range)**	**Median (range)**
Chief complaint	5 (4–5)	5 (4–5)
History of present illness	5 (4–5)	5 (2–5)
Physical examination	4 (3–5)	4 (3–5)
Mental status examination	3 (2–5)	5 (2–5)
Laboratory tests	4 (3–5)	4 (2–5)
Traditional healing examination	3 (1–5)	3 (1–5)
Root causes	Median (range)	Median (range)
Biological	5 (2–5)	5 (2–5)
Psychological	5 (3–5)	5 (4–5)
Social	4 (3–5)	5 (3–5)
Spiritual	3 (2–5)	4 (2–5)

### Motivation to use VPs for training

Table 2 shows results of self-reports of motivation to use the VP before and after the simulation exercise. There were no significant changes between the pre- and post-tests. Interestingly, many of the questions regarding the use of this type of VPs for training the management of traumatized patients were answered with rather high values, even in the pre-test (questions 9, 11, 13 & 14). Further on, very few of the PCPs indicated that they believed that they could meet more patients per hour (Q 5) after training with the VP, or that this case only was good to manage Bosnian refugees (Q16), which both are seen as positive.

**Table 2:** Self-reports of current motivation (pre-test questionnaire and post-test questionnaire) ranked by level of emphasis (1 = highly disagree; 4 = highly agree)

**Variables**	**Pre-test**	**Post-test**
	**All (N = 11)**	**All (N = 11)**
**Collected data**	**Median (range)**	**Median (range)**
1. I am motivated to use VP as it leads to better care	3 (3–4)	4 (2–4)
2. I am motivated to use VP as I will feel more competent	3 (3–4)	4 (1–4)
3. I am motivated to use VP as I will have a better relationship with the patient	3 (2–4)	3 (1–4)
4. I am motivated to use VP as it will provide better treatment outcomes	3 (3–4)	4 (1–4)
5. I am motivated to use VP as I can meet more patients per hour	2 (1–3)	1 (1–2)
6. I am motivated to use VP as I can have more time with the patient	3 (1–4)	3 (1–4)
7. I am motivated to use VP as I can use VP to educate my staff	3 (2–4)	4 (2–4)
8. I am motivated to use VP as it helps to improve interdisciplinary communication	3 (3–4)	3 (2–4)
9. I am motivated to use VP as it helps me to understand the mental health problems of my patient	4 (3–4)	3 (2–4)
10. I am motivated to use VP as it helps me to understand medical problems of my patient	3 (2–4)	3 (1–4)
11. I am motivated to use VP as it helps me to understand social problems of my patient	4 (3–4)	3 (2–4)
12. I am motivated to use VP as it helps me to understand spiritual problems of my patient	3 (2–4)	3 (1–4)
13. I believe the VP will help me provide better care to all of my patients	4 (2–4)	3 (1–4)
14. I believe the VP will help me provide better care of my traumatized patients from any cultural background	4 (3–4)	4 (2–4)
15. I believe the VP will help me provide better care to my traumatized patients who are from culturally diverse backgrounds and are low-English speakers	3 (1–4)	4 (2–4)
16. This system is only good for helping me to manage traumatized refugees from Bosnia	1 (1–4)	1 (1–4)

### General opinions and perceptions in the questionnaires after the learning experience

Most of the participants were positive to the VP as being a great and helpful learning tool for exercising skills that appeared realistic and interactive. Some felt that it helped to expand the thinking in “Domains”.

- *It [VP] appeared real. When I started the program and was trying to figure out what to press, Katharina (VP) started to cough as a sign that she was waiting for me to start. I thought that was interesting.*

After the simulation exercise, the participants were more concrete in how to use VP in the future. They found VPs to be a great teaching tool and very valuable not only for medicine but for any healthcare discipline. They considered that it was user-friendly and it could not only be used to assess patient’s health but also patient’s resources. There were also critical comments.

- *I liked it. It’s a great learning tool, many things can be done with it. Listening/watching a real person add a lot to the experience.*

- I can’t think of any reasons why we should be using (this). There are better reasons to use it to improve our patient’s quality of regarding critique. Thu, its will improve our knowledge.

Regarding the participants’ perceptions of virtual patients as compared to paper cases (Likert scale ranging from worse to better), seven of the 11 participants answered Median = 5 (better).

- *Emotions, tone of voice, body language enhanced the experience [of VP].*

The overall opinion about this learning method was positive: it was perceived as an easy and safe way to train relevant skills, good at various levels (beginners/medical students & experts/residents) and gave an opportunity to learn and refresh their interviewing skills of a full assessment and at the same time observe the patient’s reaction and response to their questions. Compared to hearing a lecture or reading relevant literature, VP was perceived as better. The video quality of the VP was mentioned to be good. Some recommended to have a summary in the end of the personal story *“to point out a few general facts about how to approach others who have suffered trauma.”*

- *When first working with people who are victims/survivors of trauma a clinician can be overwhelmed to the point of that they themselves being traumatized. Through this learning method, the clinician can feel those moments within themselves, learn to deal with them, put the aside to a more appropriate place/time to talk about them* etc. *But initially those “Oh my God” moments maybe hard to deal with appropriately.*

The physical examination feature was evaluated as helpful by the participants but for some of them it was not considered as enough, as they wanted to pay more attention to gynecological problems as well as reflexes and range of motions in arms, than they could. An additional limitation mentioned was that the participants could not physically touch the patient in order to i.e. auscultate her lungs or heart sounds, or feel her pulse. But they still felt it valuable to be able to watch the patient’s reaction:

- …*her frustration with waiting for the next question, her body language, her tone of voice all were key aspects to the assessment–certainly it added to the learning experience.*

Few of the participants felt that VP was an “ideal real patient”, and directed mostly to her psychological problems and reported this experience as follow:

- *Most people I see are further out from their refugee/trauma experiences than this patient, and less likely to have semi-acute medical issues like gonorrhea.*

Others felt that after being trained with the VP, they became more open to ask people about their stories and to have full assessment of the patient’s status–physical, emotional, social and spiritual, which reinforces the complexity of patients.

It was common that the participants wanted to have the possibility to formulate their own questions in free text and experienced that scrolling among the pre-defined illness history questions in order to find the most suitable question was in some way odd. Some felt that the VP tool was somewhat artificial due to the limited options to ask questions in the order they wanted. They also reported that the feedback of the VP and VA was not totally clear and needed to be technically improved.

All of the 11 participants gave constructive comments to improve the design of the VP system. Their suggestions included the possibility of asking their own questions without the need of scrolling among pre-defined questions, the technical improvement of feedback by the VP and Virtual Advisor and adding more extensive history details such as information about the patient’s daughter. Moreover, a more user-friendly interface that guides the user during the beginning and the ending of the case was suggested. One of participants summarized it like this:

- *Several issues: (1) Traditional (folks) folder was empty; (2) Did not like read to scroll the questions; (3) unable to access background info–it did not open anywhere. Also the ending was confusing. I did put my assessment and submitted it, but did not get feedback. Then pressed wrong button and case was over. May be a warning “It would end your session, are you sure? Or would you like to hear feedback?*

### Feedback during the follow up interview regarding VP as authentic, acceptable, and ease to use

The participants recalled VP as a realistic and relevant virtual interface (“realistic responses to the questions”) with an interesting story that was very well done. Some remembered that the questions were very good and especially liked the four patient management domains. One participant hoped that this version could be rolled out for other languages and cultures, as it had great potential; the resource section needs a lot of work.

One of the participants remembered the VP as a useful tool especially for medical students but not for practicing doctors, as it was experienced as too artificial. It reminded some that most of their patients have some trauma in their lives and thought in a real patient would have had trouble dealing with all of that material.

- I think it was a very sad story, I feel compelled by all her losses. I hear similar stories from my patients and this story confirms my experience.

- I was moved by her. She reminded me of many of my patients.

A common answer from the participants (8 out of 10) was that VP could be used as an examination tool on many levels of competence, since it eliminates a lot of biases as everything which is used with this VP is the same.

- I think that my feeling is… it could eliminate a lot of biases because that is used with this VP is the same so the learner gets organized to think through the case.

- It gives you some distance to consider best questions and what directions should I go. The physical examination part one can’t do too much.

The participants mentioned that they would pay more attention to their interview skills after this training and that they would be more sensitive to the patient story and how emotional, psychosocial impact affects their health. They had increased awareness of the continuing effect of past trauma and were more willing to explore this concept deeper and not going too fast to traumatize the patient more. To get mastery with the four domains was also mentioned. One of the patients explored this in the following way:

- *I am more aware of listening more attentively to patients.*

The majority of the participants answered that they would recommend the VP to a colleague as a training tool in assessing a refugee trauma patient.

Suggestions for improvement of the system were given by the participants also during the follow up interviews. They wanted it to be more user friendly, for instance giving the possibility to ask their own follow-up questions. The VA should be more easily accessible and more interactive and give more feedback about the participants’ interviewing skills. Further suggestions were that physical examination module needs to be expanded or should be skipped and some of the information was a little disjointed. It was recommended that the flow of questions should be increased and made more natural and with less structure. The participants would also have liked to be informed that it was not obligatory to ask all of the questions provided by the system.

- The patient and the answers were very real life. It was clear that a lot of work was put into it and it was very believable. However, the questions and the system were not easy to use with the click and submit methods.

## Discussion

The main aim of this study was to present results from developing and evaluating a virtual patient simulation for training the management of traumatized refugees and how the VP system was perceived by PCPs. In summary, this study demonstrated that the participants’ responses were positive and this VP system was seen as a promising educational tool for increasing their knowledge in the area of caring for patients with trauma, mental illness and refugee background. Other positive comments paid attention to being more relevant than a paper case in the training of medical students. The participants ranked the mental status examination more positive after the simulation exercise compared to before the simulation. Only minor changes was found in datapoints between the pre- and post-tests of self-reported motivation to use VP as an educational tool. Although the general motivation as ranked during the pre-test was high, the participants exhibited high expectations and a positive attitude towards the VP system. Some items in post-test scored, however lower in the post-tests (including items 9, 11 and 13) which can be interpreted as that the participants acquired a more realistic view on the current version of our VP having it’s limitations and areas that need improvement. The results were supported by the follow up interviews. Comparing answers to motivation in the questionnaire versus telephone/face-to-face follow up, most are moving in the same direction. In terms of validity, this pilot study also favors the system’s face validity as the participants had a positive attitude to the VP system which was also found in the study by Pantziaras et al. [43].

In complex patients with multiple problems and poor communication skills such as refugee patients, traditional PCP approach is often inadequate. Siebens [56] has responded to these limitations by creating a systematic approach to organizing the PHC use of clinical information called the Domain Management Model (DMM). The DMM provides a standard approach and language to the entire clinical care process consistent with the principles of evidence and culture-based medicine. The DMM is a practical application of the biopsychosocial approach first described by Engel [57] and primarily used by behavioral health clinicians. Due to the results in this study, that the participants had increased awareness of the continuing effect of past trauma, we recommend an additional domain, a trauma domain in a forthcoming RCT as the PCPs maybe will not pay attention to severe traumatic life events in the assessment and follow up of the patient with trauma.

Even though virtual clinical encounters are quite a new paradigm in PHC, the participants in the present study considered our VP prototype to be a relevant and promising educational tool. The participants also experienced VP as a real clinical assessment tool, which let them use their clinical competence.

Most of the respondents suggested that the VP case may also be useful for exams. This is positive and something that is used as a routine nowadays at both medical schools and as board exams cf. [37,38]. This also indicated that the PCPs in our study believe the RTSim system is capable of visualizing cases that resemble real patient cases.

However, the most common application of VPs is for learning and training clinical reasoning. Our VP educational model based PHC imply a cognitive constructivist pedagogy and a situated learning approach [29,58] which may promote medical students’ patient-centered skills in interviewing for assessment, diagnosing and follow up. Good patient-centered interviewing skills have been connected with improved health outcomes and these strategies can according to Lein and Wills [59] “enhance effectiveness of patient care processes and outcomes while retaining efficiency of patient management” (page 215). Previous research has pointed out that VPs cases need to be realistic and preferably also based on real cases [60-62]. The more authentic a virtual patient case is, it is more challenging [61], which may explain some of the reservations expressed by one of the participants who remembered the VP as a useful tool especially for medical students but not for practicing doctors, as it was experienced as too artificial.

The reduction in post motivation scores in items 9, 11 & 13 seems to show that the VP training was negative in some aspects, mainly because of the high expectations before the training procedure. This is called expertise reversal effect, i.e. the relative challenges in expert learners’ performance may happen “when there are overlaps between their well-learned and proceduralized knowledge structures and provided instructional guidance” [63], p. 333. This evidence has significant implications for research in educational theory for adult, experienced learners as compared with novices, like medical students.

### Practical implementation of VPs for clinical training

This study investigated how a small number of PCPs at a local clinic could make use of VPs for training their skills and even though none of them had used virtual patients before, they seemed to be able to run the RTSim system without any major problems. This indicates that VPs also might be used in clinical settings, where normally no teachers or facilitators are available. Therefore, we interpret our results that VPs might be used in for example CME in also other settings without too much need of support. This is in line with previous research [61], where CME has been indicated as one potential use of VPs.

The practical implementation of VPs for training has also been discussed in other studies, and indicated that it might be advantageous to use VPs in PBL settings or in other small groups as pairs of learners [64]. However, in clinical settings and/or for CME, individual use is probably the method of choice due to limitation in time and space for clinicians with heavy clinical burdens.

### Limitations

One of the limitations in this study was the rather small N, which did not allow for generalizability of gender and age, which may have some impact on the PCPs’ perceptions of the utilization of the VP in PHC as suggested by the initial study [34].

The results were based on the subjective experiences of the participants without any external video observations, physiological recordings or other objective data [64] which could more deeply pay attention to the participants’ thoughts and attitudes to the VP.

There were some limitations due to technical issues which may have influenced the motivation of using the VP. Positive findings from the questionnaires included that the PCPs put more focus on mental status examination after the VP-based training. This is very positive since traumatized refugees often have mental impairment that needs to be identified, understood and taken into consideration by the PCP. Other non-significant, but still interesting positive findings included that the self-reported dimensions of clinical care showed positive trends in the post-test indicating that the PCPs tended to put more emphasis on root causes (social and spiritual) after the VP-training.

On the other hand, negative fluctuating datapoints in results came from the post-test in terms of that the participants disagreed more to use VP to help them to understand the mental health problems in their patients and to use VPs to help them to understand spiritual problems of their patients. These negative fluctuating datapoints might be connected with someone’s wish to apply open ended free text questions in order to build trust, which was not possible in the current version of the RTSim system. Such a comment was also given by one of the interviewed participants, who said that he lacked a feature to ask open ended questions to the Virtual Refugee. However, such VPs systems with free text dialogues are very expensive to develop, even with modern techniques.

## Conclusions

The need for improvement of training of PCPs in the management of traumatized refugee patients with physical and psychiatric impairment is substantial. Even though virtual clinical encounters are quite a new paradigm in PHC, the participants in the present pilot study considered RTSim to be a relevant and promising educational tool for PHC. The participants also experienced VP as a realistic clinical assessment tool, which let them use their clinical competence. The next stage of our project, Phase 2, will be to test the impact of the VP prototype compared to a paper case on improving clinical diagnosis and treatment of the traumatized refugee patient. Then we plan a RCT study to test the impact of data outcome.

## Competing interests

The authors declare that they have no competing interest.

## Authors’ contributions

SE, RM, UF, IP and JL have all made substantial contributions to conception and design of the study. SE, RM, UF and JL have been involved in the grant application. SE, RM, UF and JL acquired the data and completed data analysis, and all authors were involved in data interpretation. SE conducted the data analysis and drafted the manuscript. All authors revised it critically for important intellectual content and have given final approval of the version to be published.

## Authors’ information

Professor Richard F Mollica, MD is foreign adjunct professor at Cultural Medicine Unit, Department of Learning Informatics, Management and Ethics, Karolinska Institutet, Stockholm, Sweden (2010-05-10—2016-05-09).

## Pre-publication history

The pre-publication history for this paper can be accessed here:

http://www.biomedcentral.com/1472-6920/13/110/prepub
